# Intrahepatic cholangiocarcinoma with gastric metastasis misdiagnosed as primary gastric cancer: A case report and literature review

**DOI:** 10.3389/fonc.2022.997735

**Published:** 2022-09-05

**Authors:** Qingshun Zhu, Shengyong Zhai, Enkang Ge, Lei Li, Xuguang Jiao, Jinqiu Xiong, Guangxu Zhu, Yuanyuan Xu, Jianjun Qu, Zhengjiang Wang

**Affiliations:** ^1^ Department of Clinical Medical College, Weifang Medical University, Weifang, China; ^2^ Department of General Surgery, The first affiliated Hospital of Weifang Medical University (Weifang People’s Hospital), Weifang, China

**Keywords:** intrahepatic cholangiocarcinoma, metastasis, gastric, PET/CT, immunohistochemical staining

## Abstract

We describe a case of intrahepatic cholangiocarcinoma with gastric metastasis misdiagnosed as primary gastric cancer. In addition, combined with the literature, we summarized the clinical and imaging features of gastric metastasis of intrahepatic cholangiocarcinoma in order to improve the understanding of the preoperative diagnosis. Positron emission tomography/computed tomography (PET/CT) is accurate in evaluating the primary tumor, lymph node metastasis, and distant metastasis of patients. In addition, immunohistochemical staining can determine the primary site of metastatic adenocarcinoma. For patients who can not determine the location of the primary tumor, the rigorous preoperative examination is necessary, it can improve the accuracy of diagnosis and avoid excessive treatment of patients.

## Introduction

Intrahepatic cholangiocarcinoma mainly metastases through direct invasion, often occurs through intrahepatic metastasis, advanced distant metastasis mainly occurs in the lung, bone, brain, and other organs, but intrahepatic cholangiocarcinoma gastric metastasis is very rare. Here, we report a patient who was misdiagnosed with primary gastric cancer with gastric metastasis of intrahepatic cholangiocarcinoma treated in our hospital. As far as we know, in the English literature that can be retrieved by PubMed, only 4 cases of gastric metastasis of intrahepatic bile duct adenocarcinoma have been reported. This article combines a case of intrahepatic bile duct adenocarcinoma with gastric metastasis and reviews the related literature, hoping to attract more attention to this kind of disease in clinical practice.

## Case presentation

**Figure 1:**
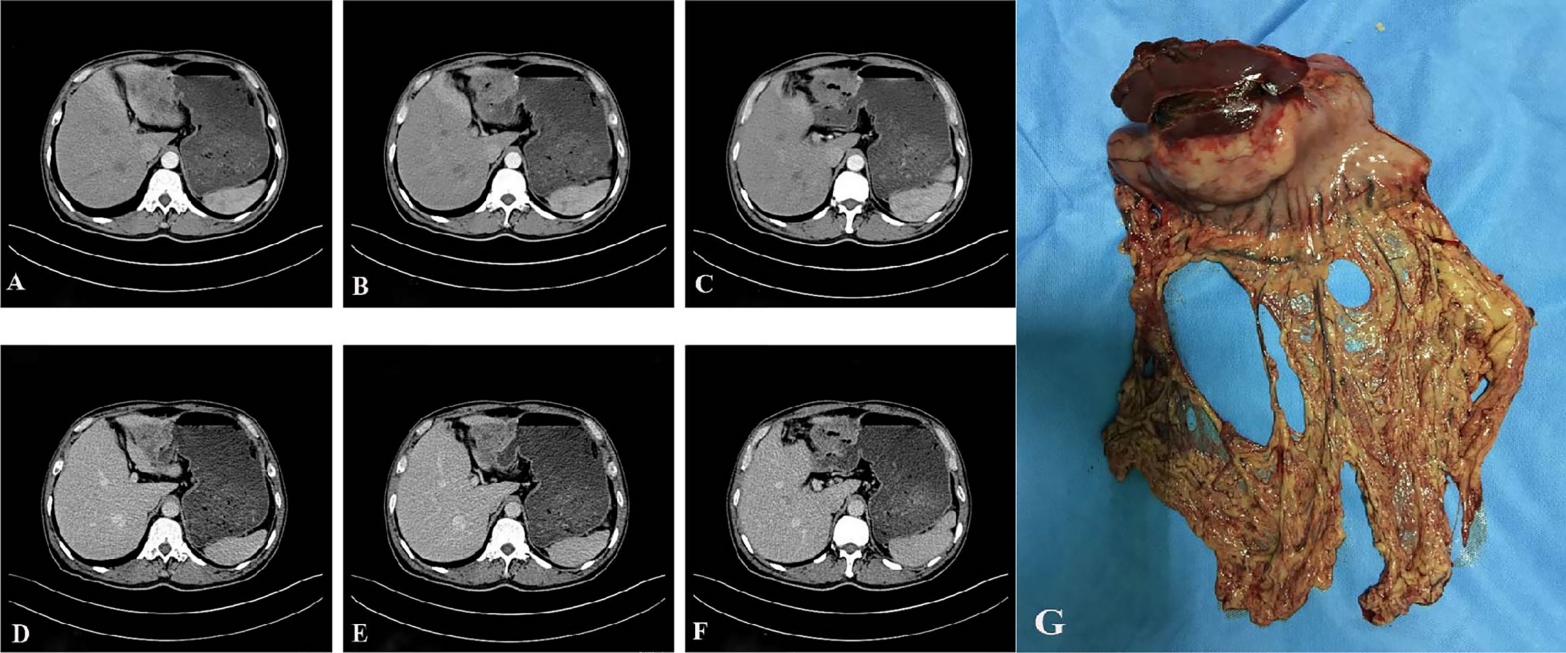
Preoperative auxiliary examination and postoperative specimen. **(A–F)**, abdominal enhanced CT showed gastric cancer thickening in the gastric antrum, and the tumor was not clearly demarcated from the left lobe of the liver; the size and shape of the liver were normal, the edge was smooth, and no abnormal density and enhancement foci were found in the parenchyma **(A–C)** shows the arterial phase; **(D–F)** shows portal venous phase). **(G)**, the distal stomach and part of the left lateral lobe of the liver after the operation.

A 57-year-old male patient presented to the local hospital in July 2017 for “epigastric pain and discomfort for one month”. Gastroscopy showed a large ulcer in the gastric antrum, and biopsy pathology showed poorly differentiated carcinoma, tending to poorly differentiated adenocarcinoma. The patient was admitted to our hospital for surgical treatment. Physical examination showed that there was only mild tenderness in the upper abdomen without rebound pain, no palpable abdominal mass, no obvious jaundice in the skin and sclera, and no obvious enlargement of superficial lymph nodes. Serological examination showed that total bilirubin (TBILI) decreased (1.8umol/L, normal range 7.4-24.1umol/L), hemoglobin (HGB) decreased (93g/L, normal range 120-160g/L), carbohydrate antigen (CA) 724 increased slightly (7.66U/ml, normal range 0-6.9U/ml), carcinoembryonic antigen (CEA), CA19-9, CA125, alpha-fetoprotein (AFP) were all in the normal range. Thorax and abdomen plain scan and contrast-enhanced CT (**Figures 1A** **–F**) showed gastric cancer thickening in the gastric antrum, considering the tumor, and the tumor was not clearly demarcated from the left lobe of the liver; the size and shape of the liver were normal, the edge was smooth, and no abnormal density and enhancement lesions were found in the parenchyma. Small lymph nodes could be seen in the hepatogastric space, but no obvious enlarged lymph nodes were found in the retroperitoneum. We initially diagnosed it as gastric antrum malignant tumor. After multidisciplinary tumor consultation, we decided to carry out surgical treatment.

**Figure 2:**
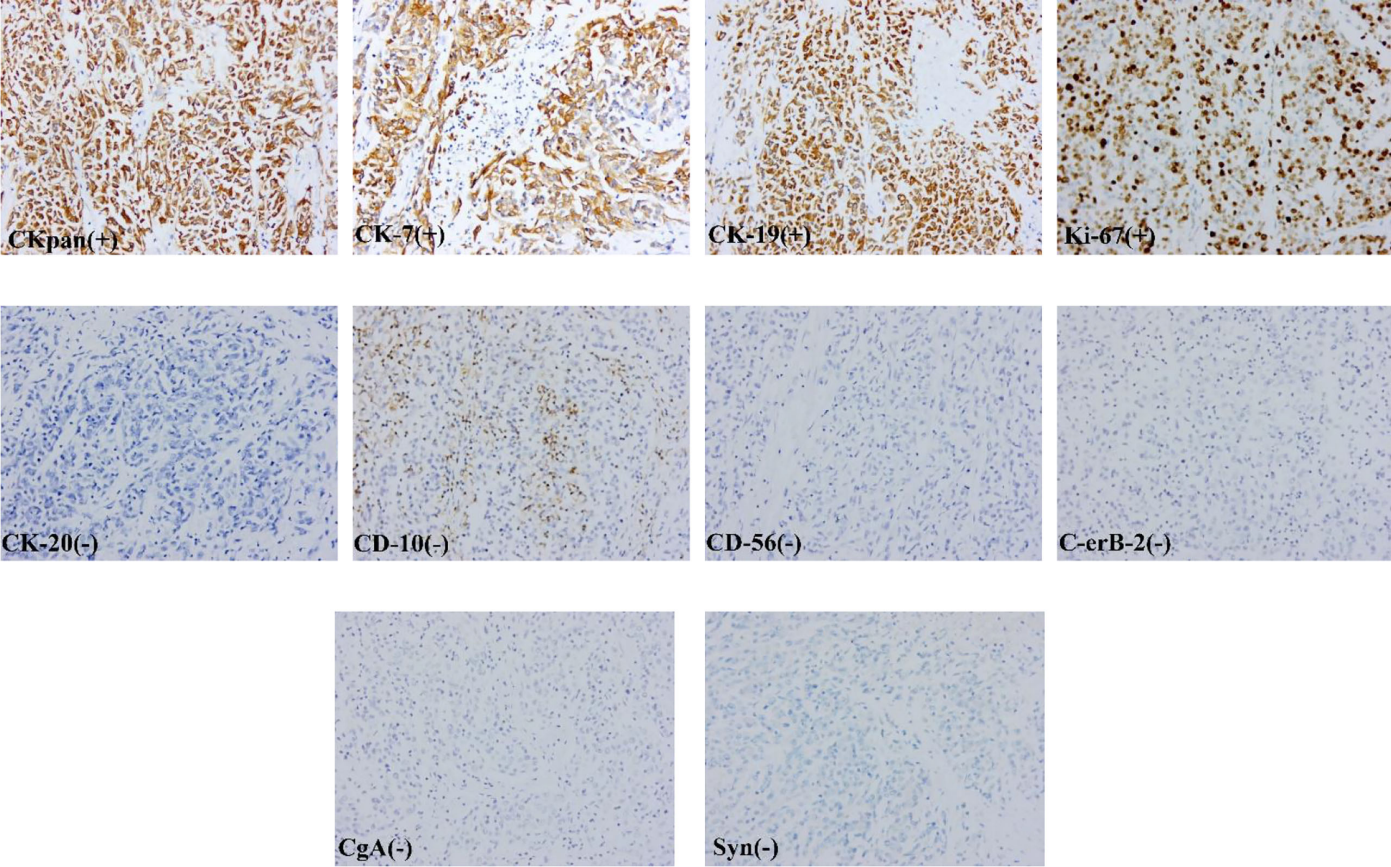
The tumor tissue expressed broad-spectrum cytokeratin (CKpan), CK-7, and CK-19, but not CK-20, C-erB-2, CD-10, CD-56, synaptophysin (Syn), chromogranin A (CgA).

After excluding surgical taboos, the patient underwent surgery in August 2017. During the operation, the tumor was located in the gastric antrum, about 5 × 4cm in size, infiltrated into the serosa, the anterior wall adhered closely to the left lateral lobe of the liver, and the posterior wall adhered closely to the transverse colon. Enlarged lymph nodes could be seen around the tumor, but there were no obvious metastatic nodules in the liver, peritoneum, and transverse colon. The family members and trustees of the patients were informed of the intraoperative findings, the details of possible adverse prognoses, and the advantages and disadvantages of different surgical methods. After obtaining their consent and signing the informed consent form, we performed radical distal gastrectomy (Billroth II gastrointestinal reconstruction) and partial left lateral lobectomy of the liver (**Figure 1G**). The operation time was about 200min, the blood loss was about 200ml, no blood transfusion was performed. The postoperative pathology showed that the tumor was poorly differentiated adenocarcinoma. The tumor was closely related to the liver tissue, invading the entire layer of the gastric wall to the mucosa from outside to inside and accompanied by ulcer formation. Tumor cells could be seen in the lymphatic vessel, and the nerve was not invaded. 27 lymph nodes were dissected. None of these lymph nodes metastasized. Immunohistochemical staining showed that the tumor tissue expressed broad-spectrum cytokeratin (CKpan), CK-7, and CK-19, but not CK-20, S-100, CD-10, Heppar-1, CD-56, synaptophysin (Syn), chromogranin A (CgA), and Ki67 proliferative index was approximately 70% (**Figures 2**). After communicating with pathologists and considering the immunohistochemical results, we considered gastric metastasis from intrahepatic biliary adenocarcinoma.

The patient recovered smoothly without obvious postoperative complications and was discharged 11 days after the operation. We performed 8 cycles of chemotherapy with the gemcitabine + capecitabine regimen. The first chemotherapy begins one month after the operation, followed by the next cycle of chemotherapy at an interval of 21 days. The patients were reexamined every 3 months after the operation for 2 years and every 6 months after the operation for 3-5 years. No tumor recurrence or metastasis was found 5 years after the operation, and the patient is still alive.

## Discussion

This patient was initially diagnosed as a malignant tumor of the gastric antrum, but according to the results of immunohistochemical staining, the diagnosis was modified to gastric metastasis of intrahepatic bile duct adenocarcinoma. Cholangiocarcinoma is a malignant tumor derived from bile duct epithelial cells, which can be divided into intrahepatic cholangiocarcinoma, hilar cholangiocarcinoma, and distal cholangiocarcinoma according to the location of the tumor (1). Intrahepatic cholangiocarcinoma is a malignant tumor originating from bile duct epithelial cells above the secondary bile duct. it is the second largest primary liver malignant tumor after hepatocellular carcinoma, accounting for 5% and 30% of primary liver cancer (2, 3). The early onset of intrahepatic cholangiocarcinoma often lacks typical clinical symptoms, most patients are often in the advanced stage and often accompanied by lymph node and surrounding organ metastasis. According to related literature reports, intrahepatic cholangiocarcinoma mainly metastases through direct invasion, often intrahepatic metastasis and advanced distant metastasis mainly occurs in the lung, bone, brain, breast, colon, skin, and blood system (4–6). Metastasis from intrahepatic cholangiocarcinoma to the stomach is very rare in clinical practice. As far as we know, in the English literature that can be retrieved by PubMed, only four cases of gastric metastasis of intrahepatic bile duct adenocarcinoma have been reported (**Table 1**). In this paper, we combined a patient with gastric metastasis of intrahepatic cholangiocarcinoma, and reviewed the related literature, hoping to attract more attention to this kind of disease in the clinic.

**Table 1:** Summary of clinical data of gastric metastasis of intrahepatic cholangiocarcinoma.

Case	Sex	Age (years)	Clinical manifestation	Primary pathology	Treatment	Survival (months)	Reference
1	Female	67	Epigastric pain	Poorly	Resection	NA	Kim EM, et al. (7)
2	Male	58	Dysphagia	Poorly	Resection	Died (5 months)	Wang C, et al. (8)
3	Female	77	Epigastric pain	Poorly	Resection	Died (5 months)	Matsuo S, et al. (9)
4	Male	80	Jaundice	Poorly	Resection	Alive (12 months)	Imamura N, et al. (10)
5	Male	57	Epigastric pain	Poorly	Resection	Alive (5 years)	Present case

Follow-up since the operation; poorly, poorly differentiated adenocarcinoma; NA, no data available.

Ultrasonography (US) is a simple and widely used non-invasive method for the diagnosis of cholangiocarcinoma. Cholangiocarcinoma often shows hypoechoic or moderate echo mass, which can be distinguished from bile duct stones. The US can also judge the blood flow signal in the tumor and whether the tumor invades blood vessels. Endoscopic ultrasound has high resolution and is not disturbed by gas, so it can directly observe the lesions of the duodenal papilla and show the structure and focus of the bile duct wall more clearly. In addition, the help of ultrasound can also locate the tumor, combined with fine needle aspiration biopsy to determine the nature and source of the tumor. CT and magnetic resonance imaging (MRI) is the preferred imaging methods for the diagnosis of intrahepatic cholangiocarcinoma. On contrast-enhanced CT, intrahepatic cholangiocarcinoma can be distinguished from hepatocellular carcinoma. On abdominal contrast-enhanced CT, irregular masses around intrahepatic cholangiocarcinoma can be seen, accompanied by hepatic lobe atrophy and local intrahepatic bile duct dilatation. Because intrahepatic cholangiocarcinoma receives blood supply from the portal vein, and cholangiocarcinoma is mostly sclerotic, with more fibrous tissue, it shows venous phase or delayed phase enhancement on CT, while hepatocellular carcinoma gets blood supply from the hepatic artery and shows arterial phase enhancement on CT (11). Cholangiocarcinoma was characterized by low signal intensity on T1-weighted and high signal intensity on T2-weighted images. The dynamic contrast-enhanced scan showed enhancement around the delayed phase. MRI is of high value in preoperative staging, resectable evaluation, selection of surgical methods, and evaluation of prognosis of cholangiocarcinoma. Li reported that the combination of enhanced CT and MRI in the diagnosis of cholangiocarcinoma has high sensitivity and specificity, which can provide an imaging basis for clinical diagnosis and treatment (12). In addition, studies have reported the dense accumulation of the nucleotide tracer 18-fluorodeoxyglucose (^18^F-FDG) in cholangiocarcinoma (13). PET scan combined with FDG accumulation can show cholangiocarcinoma as small as 1 cm of the bile duct, and it is considered that ^18^F-FDG-PET and PET/CT are accurate in evaluating primary tumor, lymph node metastasis, and distant metastasis in patients with intrahepatic cholangiocarcinoma (14, 15).

Tumor markers are rarely expressed in normal tissues, but increased in tumor tissues and blood of tumor patients, which can reflect the occurrence and development of the tumor and the situation of recurrence and metastasis. CA19-9 and CEA are elevated in 40% and 85% of patients with cholangiocarcinoma, respectively, and these markers may indicate postoperative recurrence and metastasis (16). When CA19-9 and CEA were 176.3 IU/mL and 9.6 ng/mL respectively, CA19-9 and CEA could predict the prognosis of Overall Survival (OS) (17). However, there is no recommended reference value for tumor diagnosis in the world. Although the development of science and technology is getting faster and faster, it is still difficult to diagnose cholangiocarcinoma by hematology or imaging examination. cytological and pathological examination is the gold standard for the diagnosis of cholangiocarcinoma at present. however, it is difficult to distinguish the histological manifestations of intrahepatic bile duct adenocarcinoma from metastatic non-hepatic primary tumors. Based on cytology, combined with fluorescence *in situ* hybridization (FISH) cell analysis, the specificity of diagnosis was improved. The FISH analysis uses fluorescence-labeled DNA probes to detect specific chromosomal abnormalities. A large number of special chromosome abnormalities detected by FISH can complement the diagnosis of cholangiocarcinoma (18). However, in patients affected by primary sclerosing cholangitis and biliary stricture, biopsy samples are usually insufficient for molecular spectrum analysis. in addition, tissue sampling reports have high specificity but low sensitivity in the diagnosis of malignant biliary strictures. Finally, the high embryogenic property of BTC limits the accuracy of cytological and pathological methods. In this case, fluid biopsy has attracted more and more attention. Studies have shown that fluid biopsy has great potential in the early diagnosis of cancer, the identification of driver changes, the monitoring of treatment response and the detection of drug resistance mechanisms (19, 20). Therefore, it is necessary to do more research in the field of biomarkers and further identify the specific tumor markers of cholangiocarcinoma.

Compared with other organs, the stomach is a rare site of tumor metastasis. Secondary gastrointestinal tumors are defined as primary tumors that originate outside the gastrointestinal tract or are not continuous with primary tumors in other parts of the gastrointestinal tract. Through literature search, we summarized the rare primary sites of secondary gastric tumors (**Table 2**). We found that the survival time of patients with gastric secondary malignant tumor is often very short, unlike other cases, this patient still did not find tumor recurrence and metastasis 5 years after the operation. We think this report is special and worthy of study.

**Table 2:** Rare primary sites of secondary gastric tumors.

Case	Sex	Age (years)	Primary site	Histological type	Treatment	Outcome	IHC positive marker	Reference
1	Female	73	Breast	Invasive lobular carcinoma	Resection	Bone metastases (4 months)	Mammaglobin, ER	Gurzu S, et al. (21)
2	Female	67	Breast	Invasive ductal carcinoma	Resection	Died (5 months)	CK7, CDX2, E-cadherin, SLUG, CD44	Gurzu S, et al. (21)
3	Female	82	Skin	Malignant melanoma	Radiotherapy	Died (3 months)	Melan A, HMB45, SOX-10, S100	Yoshimoto T, et al. (22)
4	Male	55	Lung	Non-small cell lung cancer	Resection	Died (3 months)	NA	Shih-Chun C, et al. (23)
5	Female	51	Ovarian	Ovarian carcinoma	Resection	NA	PR, ER, (CK7), Wilms’ tumor-1	Liu Q, et al. (24)
6	Female	70	Renal	Clear cell renal cell carcinoma	Resection	No recurrence (4 months)	CD10, CAIX	Koterazawa S, et al (25),
7	Male	71	Adrenal gland	Adrenocortical Carcinoma	Resection	Died (12 months)	Vimentin, Inhibin, Synaptophysin, NSE	Kovecsi A, et al. (26)

Follow up since the treatment; ER, estrogen receptor; PR, progesterone receptor; CK7, cytokeratin 7; NSE, neuron-specific enolase; NA, no data available.

Intrahepatic cholangiocarcinoma often invades the adjacent bile duct and liver parenchyma through direct infiltration. Distant metastasis often occurs in the lung, bone, brain, and other organs, but intrahepatic cholangiocarcinoma gastric metastasis is very rare. This raises a question that needs to be considered: how intrahepatic cholangiocarcinoma metastases to the stomach. The answer to the question of direct invasion, lymph node metastasis, hematogenous metastasis, or multiple pathways remains to be discussed. We believe that the possibility of direct invasion is greater, because the primary tumor is located in the left lateral lobe of the liver, and the rear is in direct contact with the anterior wall of the stomach. When the tumor penetrates the visceral peritoneum, it is easy to cause metastasis of adjacent organs outside the liver. Secondly, in our case, cancer cells were found in the lymphatic vessels of the patient, which may not rule out the possibility of lymph node metastasis in addition to direct invasion.

At present, for the treatment of intrahepatic bile duct adenocarcinoma, radical surgery is still the only possible cure. The 7th edition of the American Cancer Federation (AJCC) staging manual recommends routine lymph node dissection for intrahepatic cholangiocarcinoma because it is helpful for accurate staging and prognosis evaluation (2, 27, 28). However, for most patients with cholangiocarcinoma, distant metastasis occurs at the time of the initial symptoms. For patients with advanced or unresectable diseases, local and systemic chemotherapy is the main treatment choice. their goal is to control local tumor growth, alleviate symptoms, improve and maintain quality of life. Among the local treatment methods, the best evidence and most promising results are transarterial radiation embolization (TARE), hepatic arterial infusion (HAI), transarterial chemoembolization (TACE) and radiofrequency ablation (RFA). Among them, RFA is considered to be a good way to control the progression of local tumors, and the postoperative complications are low (29). In addition, comprehensive genome sequencing has determined the genetic pattern of each cholangiocarcinoma subtype. Therefore, promising molecular targets in precision medicine have been identified and are being evaluated in clinical trials, in which fibroblast growth factor receptor (FGFR) inhibitors have become a research hotspot in recent years, and futibatinib (TAS-120) has the potential to become a new treatment option for intrahepatic cholangiocarcinoma with abnormal FGFR2 (30, 31). With the use of drugs for PD-1/PD-L1 and CTLA-4, the treatment of immune checkpoint inhibitors (ICIS) has brought the treatment of cancer into a new field. Studies have shown that adjuvant therapy based on ICIS and chemotherapy has a certain guiding significance for the treatment of BTC (32, 33). At present, it is also possible to establish a preclinical model of CCA to clarify the causes and molecular mechanisms of carcinogenesis, tumor progression and metastasis; to find prognostic biomarkers and drug targets, and to test the efficacy of drugs and develop more effective treatments will also play a vital role in the development of cholangiocarcinoma treatment (34).

In our patients, we chose radical resection and postoperative adjuvant chemotherapy. At present, this treatment is beneficial to patients. However, surgical decisions should take into account the level of progress of the primary disease, and PET/CT is a very useful diagnostic tool for metastatic diseases (35). And immunohistochemical markers can determine the primary site of metastatic adenocarcinoma (36). Therefore, it is necessary to perform immunohistochemical staining on the biopsy tissue. If the location of the primary tumor cannot be determined, the removal of metastatic tumor tissue can help locate the primary tumor.

## Conclusion

Gastric metastasis of intrahepatic cholangiocarcinoma is clinically rare, and we suspect that it is related to direct invasion, but the characteristic of this patient is that no obvious tumor in the liver was found by preoperative auxiliary examination. Therefore, a detailed preoperative examination is very necessary to improve the accuracy of diagnosis. PET/CT is accurate in evaluating patients’ primary tumor, lymph node metastasis, and distant metastasis. in addition, immunohistochemical markers can determine the primary site of metastatic adenocarcinoma. At present, radical resection and postoperative adjuvant chemotherapy may be beneficial to patients. In addition, targeted therapy and immunotherapy for intrahepatic cholangiocarcinoma have also become the focus of research. It is believed that the most beneficial treatment for patients with gastric metastasis of intrahepatic cholangiocarcinoma can be determined by further research in the future.

## Data availability statement

The original contributions presented in the study are included in the article/supplementary material, further inquiries can be directed to the corresponding authors.

## Ethics statement

Written informed consent was obtained from the individual(s) for the publication of any potentially identifiable images or data included in this article.

## Author contributions

JQ and ZW: guarantees the integrity of the entire study and edited the manuscript. QZ, SZ, EG, and LL: prepared and edited the manuscript. XJ, JX, GZ, and YX: performed the literature research, data analysis, and text proofreading. All authors contributed to the article and approved the submitted version.

## Funding

This study is supported by the Medical and Health Science Technology Development Program in Shandong Province (202104080159) and the Science and Technology Development Program in Weifang City (2019YX002 and 2021YX007) and the Scientific Research Project of Weifang Health Commission (WFWSJK-2021-028).

## Acknowledgments

I would like to express my gratitude to all those who helped me during the writing of this manuscript and their efforts in the management of this patient.

## Conflict of interest

The authors declare that the research was conducted in the absence of any commercial or financial relationships that could be construed as a potential conflict of interest.

## Publisher’s note

All claims expressed in this article are solely those of the authors and do not necessarily represent those of their affiliated organizations, or those of the publisher, the editors and the reviewers. Any product that may be evaluated in this article, or claim that may be made by its manufacturer, is not guaranteed or endorsed by the publisher.
